# Mechanisms of Abrupt Loss of Virus Control in a Cohort of Previous HIV Controllers

**DOI:** 10.1128/JVI.01436-18

**Published:** 2019-02-05

**Authors:** Miriam Rosás-Umbert, Anuska Llano, Rocío Bellido, Alex Olvera, Marta Ruiz-Riol, Muntsa Rocafort, Marco A. Fernández, Patricia Cobarsi, Manel Crespo, Lucy Dorrell, Jorge del Romero, José Alcami, Roger Paredes, Christian Brander, Beatriz Mothe

**Affiliations:** a IrsiCaixa AIDS Research Institute–HIVACAT, Hospital Universitari Germans Trias i Pujol, Badalona, Spain; b Universitat Autònoma de Barcelona, Barcelona, Spain; c Flow Cytometry Facility, Health Sciences Research Institute Germans Trias i Pujol, Badalona, Spain; d HIV Unit, Infectious Diseases Department, Hospital Universitari Germans Trias i Pujol, Badalona, Spain; e Infectious Diseases Unit, Internal Medicine Department, Complexo Hospitalario Universitario de Vigo, IIS Galicia Sur, Spain; f Nuffield Department of Medicine, University of Oxford, Oxford, United Kingdom; g Centro Sanitario Sandoval, Madrid, Spain; h Instituto de Salud Carlos III, Madrid, Spain; i University of Vic and Central Catalonia, UVIC-UCC, Vic, Spain; j Institució Catalana de Recerca i Estudis Avançats (ICREA), Barcelona, Spain; k AELIX Therapeutics, Barcelona, Spain; Ulm University Medical Center

**Keywords:** HIV-1 control, HIV-1 progression, cell tropism, host genetics, *in vitro* virus inhibition, loss of control

## Abstract

Elite and viremic HIV controllers are able to control their HIV infection and maintain undetectable or low-level viremia in the absence of antiretroviral treatment. Despite extensive studies, the immune factors responsible for such exclusive control remain poorly defined. We identified a cohort of 14 HIV controllers that suffered an abrupt loss of HIV control (LoC) to investigate possible mechanisms and virological and immunological events related to the sudden loss of control. The in-depth analysis of these subjects involved the study of cell tropism of circulating virus, evidence for HIV superinfection, cellular immune responses to HIV, as well as an examination of viral adaptation to host immunity by Gag sequencing. Our data demonstrate that a poor capacity of T cells to mediate *in vitro* viral suppression, even in the context of protective HLA alleles, predicts a loss of viral control. In addition, the data suggest that inefficient viral control may be explained by an increase of CD8 T-cell activation and exhaustion before LoC. Furthermore, we detected a switch from C5- to X4-tropic viruses in 4 individuals after loss of control, suggesting that tropism shift might also contribute to disease progression in HIV controllers. The significantly reduced inhibition of *in vitro* viral replication and increased expression of activation and exhaustion markers preceding the abrupt loss of viral control may help identify untreated HIV controllers that are at risk of losing control and may offer a useful tool for monitoring individuals during treatment interruption phases in therapeutic vaccine trials.

**IMPORTANCE** A few individuals can control HIV infection without the need for antiretroviral treatment and are referred to as HIV controllers. We have studied HIV controllers who suddenly lose this ability and present with high *in vivo* viral replication and decays in their CD4^+^ T-cell counts to identify potential immune and virological factors that were responsible for initial virus control. We identify *in vitro*-determined reductions in the ability of CD8 T cells to suppress viral control and the presence of PD-1-expressing CD8^+^ T cells with a naive immune phenotype as potential predictors of *in vivo* loss of virus control. The findings could be important for the clinical management of HIV controller individuals, and it may offer an important tool to anticipate viral rebound in individuals in clinical studies that include combination antiretroviral therapy (cART) treatment interruptions and which, if not treated quickly, could pose a significant risk to the trial participants.

## Abstract (precis)

A few individuals can control HIV infection without the need for antiretroviral treatment and are referred to as HIV controllers. We have studied HIV controllers who suddenly lose this ability and present with high *in vivo* viral replication and decays in their CD4^+^ T-cell counts to identify potential immune and virological factors that were responsible for initial virus control. We identify *in vitro*-determined reductions in the ability of CD8 T cells to suppress viral control and the presence of PD-1-expressing CD8^+^ T cells with a naive immune phenotype as potential predictors of *in vivo* loss of virus control. The findings could be important for the clinical management of HIV controller individuals, and it may offer an important tool to anticipate viral rebound in individuals in clinical studies that include combination antiretroviral therapy (cART) treatment interruptions and which, if not treated quickly, could pose a significant risk to the trial participants.

## INTRODUCTION

There is a small proportion of HIV-1-infected individuals that spontaneously control HIV infection (1, 2). Due to the heterogeneity among individuals with this clinical course (3, 4) they are referred to as long-term nonprogressors (LTNP), HIV controllers, or, in the case of undetectable viremia, elite controllers. Several factors have been postulated to play a role in this viral control, including host genetic, immunological, and viral factors. In particular, host genetic markers have been associated with disease progression, yet their mechanistic action remains uncertain (5). Possibly, the strongest predictors of HIV control include polymorphisms in HLA class I alleles, which alone or in combination with killer cell immunoglobulin-like receptors (KIR) have been linked to sustained low-level viremia in the absence of combination antiretroviral therapy (cART) (6–8). Since the HLA class I-encoded gene products present virus-derived T-cell epitopes to CD8^+^ T cells, an extensive number of studies exist that have also linked T-cell responses and their specificities to HIV control (2, 9, 10). Aside from host genetics and immune factors, viral factors, such as viral replication capacity and cell tropism, have been associated with HIV control, although cell tropism has not been consistently documented (11, 12).

During the course of HIV infection, a proportion of nonprogressor individuals may suffer a disruption of their capacity to control infection, which can manifest itself in different ways, as follows: clinical progression defined as a new AIDS-defining event, immunological progression defined as an abrupt decrease of CD4^+^ T-cell counts, and/or virological progression as a significant increase in viral loads (13–16). In addition, HIV superinfection has been identified as a possible explanation for sudden signs of uncontrolled HIV infection (17). Specific plasma cytokine profiles and Gag-specific T-cell responses have been linked as well to eventual loss of control in an elite controller cohort (18). However, the contribution of these factors to a sudden loss of control is poorly defined, in part due to the scarce availability of longitudinal samples from such individuals, especially of samples close to the time point before loss of viral control (LoC). Here, we have identified and longitudinally followed 14 individuals who experienced an abrupt transition to progressive HIV infection with pre- and post-LoC samples available within 1 year, and we have integrated host, virological, and immune parameters in order to better define the mechanisms underlying the progression of HIV-1 infection.

## RESULTS

### Clinical characteristics of HIV controllers experiencing abrupt LoC.

**FIG 1:**
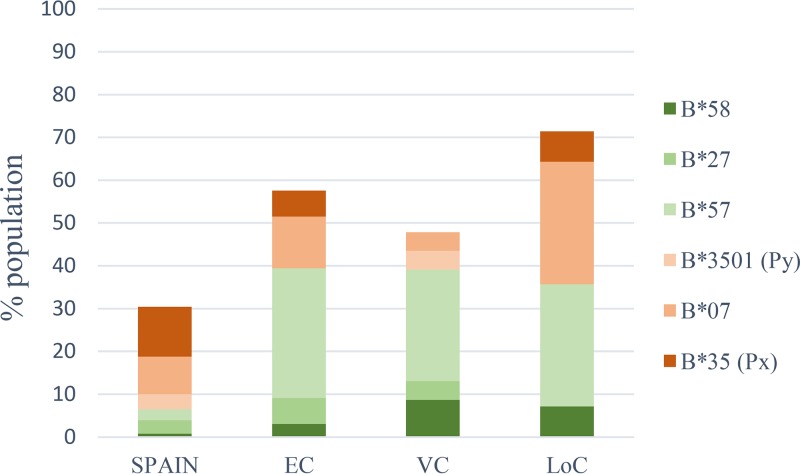
HLA class I allele frequencies. HLA-B allele frequencies are shown for the general Spanish population (64), a cohort of elite controllers (EC, *n* = 38), viremic controllers (VC, *n* = 27), and individuals that have abruptly lost their capacity to control the infection (LoC, *n* = 14).

For the present analyses, 14 individuals with LoC were identified after reviewing clinical criteria and sample availability for HIV-infected individuals with elite controller (EC) or viremic controller (VC) status from the IrsiCaixa and the BioBank Controllers cohorts (Table 1). The evolution of viral load and CD4 counts is shown in Fig. S1 in the supplemental material. Samples prior to LoC were obtained with a median of 20 months before the first signs of LoC defined either as detection of uncontrolled viral load (*n* = 4) or a concomitant raise in viral load and reduction in CD4 counts (*n* = 10, Fig. S1). Follow-up samples included samples taken at first diagnosis of LoC (*n* = 11) or within the following 6 months (*n* = 3). As HIV control has been linked to HLA class I genotypes, high-resolution HLA class I typing was performed on all 14 individuals included in the study. In line with data from other cohorts of elite and viremic controllers (19, 20), protective HLA class I alleles B57 and B58 were more common in this group of individuals (Fig. 1) than in the general population in Spain and similar to the frequencies seen in the EC and VC comparison group established at our center. Thus, in this relatively small cohort, no specific class I HLA was associated with LoC in individuals with previous HIV control.

**TABLE 1:** Clinical characteristics of the 14 controllers with loss of HIV control

ID^*a*^	Age (yr)^*b*^	Sex	Time since known HIV-1 infection (yr)^*b*^	Log_10_ of viral load at control timepoint studied^*c*^	Log_10_ of viral load at peak of loss of control	No. (%) of CD4 cells/mm^3^ at control time point studied	No. (%) of CD4 cells/mm^3^ at loss of control	HLA-A^*e*^	HLA-B^*e*^	HLA-C^*e*^	HLA-DRB^*e*^	HLA-DQB^*d*^^,^^*e*^
LP1	44	Male	16	UD	3.66	1,007 (37)	426 (18)	0201/3002	1801/5701	0501/0602	0301/0701	0201/0303
LP2	54	Female	25	3.13	5.18	721 (44)	171 (19)	0201/3402	1401/4402	0501/0802	1301/1454	NA
LP3	42	Male	3	2.74	4.36	507 (26)	265 (17)	0101/1101	3508/5201	0401/1202	0801/1502	0402/0601
LP4	23	Male	3	UD	4.90	1148 (32)	858 (29)	0201/2402	1501/4403	0303/1502	0103/1103	0301/0501
LP5	37	Male	13	2.84	5.63	495 (25)	266 (15)	0201/0301	5101/5101	1502/1502	1301/1301	1603/1603
LP6	47	Female	25	UD	3.70	438 (25)	282 (18)	0201/2902	4001/5701	0304/0602	0401/0701	0302/0303
LP7	36	Male	4	2.40	4.72	1,128 (47)	555 (31)	0101/3101	4001/5701	0304/0602	0404/0701	0302/0303
LP8	43	Female	18	3.49	5.20	534 (33)	166 (11)	0301/2501	0702/5101	0702/0303	0901/1101	0301/0303
LP9	44	Male	14	3.66	5.02	748 (21)	402 (22)	0301/6802	0702/5301	0702/0401	1301/1302	0603/0604
LP10	38	Male	13	UD	5.90	913 (32)	400 (21)	1101/2402	0702/5101	0702/1502	0803/1501	0301/0602
LP11	44	Male	14	2.96	5.36	920	464	0201/3001	4501/5701	0602/0701	0405/1502	0302/0601
LP12	45	Male	23	2.93	4.00	810	493	0201/3201	1302/1501	0303/0602	0701/1401	0202/0301
LP13	45	Male	16	2.52	3.64	434 (32)	266 (22)	0301/1101	0702/5201	0702/1202	1301/1502	0601/0603
LP14	35	Male	1	2.63	5.20	430	465	0205/2402	0801/5801	0701/0702	0301/1102	0201/0301

a ID, identifier; LP, late progressor.

b Age and HIV duration are shown for the latest time point analyzed.

c UD, undetermined.

d NA, not available.

e Individual HLA class I typing is shown (A, B, C, DRB, and DQB).

### Evidence for virus tropism change and superinfection between pre- and post-LoC time points.

**FIG 2:**
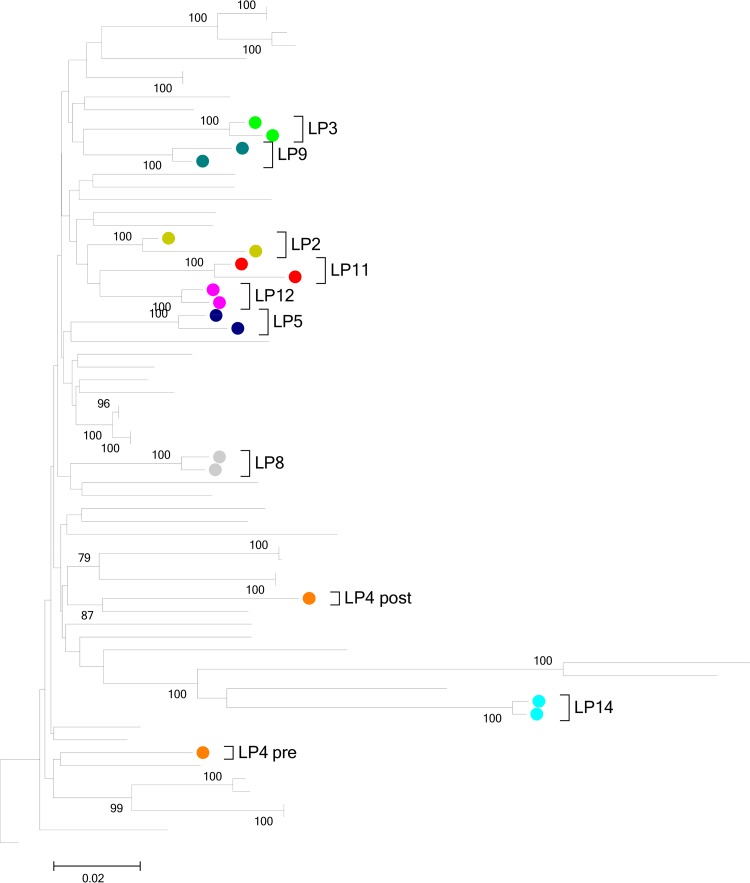
Evolutionary relationships of taxa. The evolutionary history was inferred using the neighbor-joining method (65). The optimal tree with the sum of branch length of 1.91362276 is shown. The percentage of replicate trees in which the associated taxa clustered together in the bootstrap test (1,000 replicates) is shown next to the branches (66). The tree is drawn to scale, with branch lengths in the same units as those of the evolutionary distances used to infer the phylogenetic tree. The evolutionary distances were computed using the Tamura-Nei method (67) and are in the units of the number of base substitutions per site. The rate variation among sites was modeled with a gamma distribution (shape parameter = 1). The analysis was based on a 66-nucleotide amplicon in the V3-loop of HIV gp120. All ambiguous positions were removed for each sequence pair. Evolutionary analyses were conducted in MEGA5 (68).

In order to determine whether changes in the cellular tropism of plasma virus preceded the abrupt loss of virus control, we determined viral tropism in plasma samples drawn before (*n* = 9; median, 21 months; range, 5 to 74 months) and after (*n* = 11), at diagnosis (*n* = 8), or during the peak of viremia (*n* = 3) clinical progression. Of the 14 samples obtained while the individuals had controlled infection, viral sequences covering the V3-loop (gp160) in all cases were indicative of a dominant CCR5-tropic viral population. In contrast, CXCR4-tropic plasma viruses were detected in four individuals post-LoC (although one of the individual was approaching the false-positive threshold of 10%), of which three had confirmed R5 virus pre-LoC (one did not amplify for *env*; Table 2). Based on phylogenetic analyses of Gag sequences, all pre- and post-LoC sequences showed close clustering for each individual except for one (subject LP4, Fig. 2), suggesting a superinfection event. Overall, clear evidence for changes in cell tropism were observed in one-quarter of the studied cohort, while superinfection was not a generalized mechanism for LoC in this group of patients.

**TABLE 2:** HIV tropism before and after loss of control HIV tropism was estimated by Gp120 V3-loop region sequencing before and after late progression^*a*^

ID	Control-Pre (%FPR)	LoC-Post (%FPR)
LP1	NA	CCR5 (12.5)
LP2	CCR5 (36.2)	CXCR4 (0.1)
LP3	CCR5 (35.5)	CCR5 (35.3)
LP4	NA	CXCR4 (3.2)
LP5	CCR5 (13.2)	CXCR4 (8.6)
LP6	NA	NA
LP7	CCR5 (36.2)	CXCR4 (4.8)
LP8	CCR5 (10)	CCR5 (41.4)
LP9	CCR5 (55)	CCR5 (28.8)
LP10	CCR5 (42.3)	CCR5 (28.8)
LP11	CCR5 (38)	CCR5 (37.4)
LP12	NA	CCR5 (86.5)
LP13	NA	CCR5 (42.2)
LP14	CCR5 (55.1)	CCR5 (55.1)

a The false-positive rate (FPR) was set to 10% to identify X4 tropism. NA, sample not amplified.

### Virus maintained HLA-associated escape mutations.

**FIG 3:**
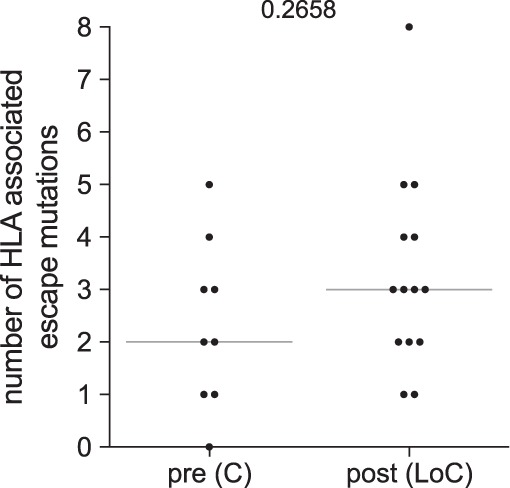
Number of HLA escape mutations in Gag (23) detected in viral population from samples before and after loss of control.

Viral breakthrough from immune surveillance has been described for both T- and B-cell-mediated immune control and could contribute to the sudden raise in viral loads and clinical HIV disease progression (21, 22). In order to evaluate whether the presence and accumulation of T-cell escape mutations contribute to LoC, HIV Gag sequences were obtained from samples drawn before and after LoC and analyzed for the presence of HLA class I footprints (23) (Fig. 2). All individuals yielded sequences in post-LoC samples, while HIV Gag sequences were successfully amplified in 9 out of 14 pre-LoC samples when subjects presented with effective *in vivo* virus control and low or undetectable viral loads. Gag sequences were compared with described HLA-associated escape mutations specific to the individuals’ HLA class I genotype (23). In 67% of patients with pre- and post-LoC sequences available, the number of patient-HLA specific footprints increased from pre- to post-LoC sequences. Despite this trend, the median number of footprints post-LoC (median, 3 footprints; range 1 to 8 footprints) did not differ significantly from the frequency of HLA footprints pre-LoC (median, 2 footprints; range, 1 to 5 footprints; Fig. 3, *P* = 0.2682). Although limited to Gag, the target of some of the most effective antiviral T-cell responses (10), and although single epitope mutations can lead to a complete loss of control, these data do not suggest that broad cytotoxic T lymphocyte (CTL) escape on multiple epitopes is the major driving force for LoC in the present cohort.

### Increased number of HIV-specific T-cell responses post-LoC.

**FIG 4:**
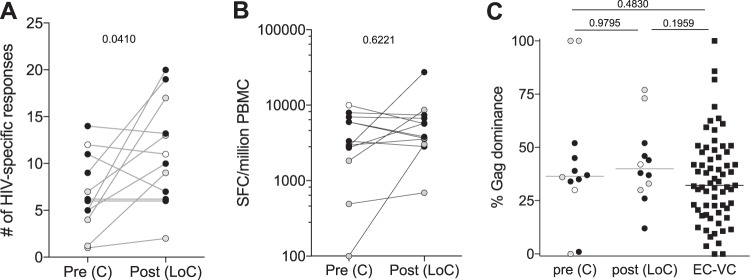
HIV-specific T-cell responses. (A) Breadth of HIV-specific T-cell responses before and after loss of control (*n* = 12). (B) Magnitude of responses, expressed as SFC/million PBMC, is shown longitudinally (C) Gag dominance (magnitude of Gag responses/total magnitude) is shown before and after evolution in the 12 LoC subjects and compared to a cohort of elite (EC) and viremic (VC) controllers (*n* = 65). Individuals that maintained an CCR5 tropism are shown in black dots, individuals that changed to a CXCR4 are shown in gray dots, and the individual from which tropism was not available is shown in white dots. *P* values are shown for Wilcoxon signed rank test in a comparison of paired data and for Mann-Whitney test to compare between groups.

Individuals that control HIV infection have been shown to have a robust HIV-1-specific CD8^+^ T-cell response, especially against the Gag protein, compared to responses observed in noncontrollers (10, 24). The HIV-specific CD8^+^ T cells responses before LoC were thus measured and compared to those of post-LoC samples and to responses seen in 65 individuals from the IrsiCaixa Controllers cohort. Responses to the entire HIV proteome were measured by gamma interferon (IFN-γ) enzyme-linked immunosorbent spot assay (ELISPOT) using a 410-overlapping-peptide set, covering the complete HIV genome, as described previously (25). Twelve out of the 14 individuals had sufficient cell viability to conduct longitudinal full-proteome HIV ELISPOT screens (Fig. 3). In nine of them, the number of individual responses remained stable (*n* = 1 individual) or increased (*n* = 8 individuals), with a significant increase in the median breadth of response from 6 responses (range, 1 to 14 responses) pre-LoC to a median of 11 responses (range, 2 to 20 responses; Fig. 4A; *P* = 0.0488). Even though the appearance of more responses post-LoC may be due to higher viral replication and antigen availability post-LoC, the increase in the breadth of responses did not result in a significant increase in the total magnitude (median magnitude from pre-LoC, 3,145 spot-forming cells [SFC]/million peripheral blood mononuclear cells [PBMC; range, 100 to 7,950 SFC/million PBMC] to median post-LoC of 4,745 SFC/million PBMC [range, 690 to 27,420 SFC/million PBMC; Fig. 4B; *P* = 0.6221]. Of note, the dominance of Gag-specific responses which characterizes naturally controlling individuals was not lost after LoC and was similar to levels tested in the comparison cohort of elite and viremic controllers (Fig. 4C andS3). Furthermore, there was no consistent pattern in the emergence of new and expansion/reduction of preexisting responses from pre- to post-LoC; however, while some individuals showed a clear loss of immunodominant responses to certain HLA-restricted epitopes in the absence of detectable mutations, other patients maintained a stable response pattern (Fig. S3). Furthermore, there were no differences in the changes in responses between individuals in whom the virus changed tropism and individuals that maintained R5-tropic virus. Interestingly, the one individual (LP4) that showed evidence of superinfection had a limited breadth of responses pre-LoC (4 responses), of which only 2 were still detectable post-LoC, while there were an additional 15 responses detected post-LoC, possibly reflecting new targets in the superinfecting virus. In this individual, viral sequence data for Gag indicated that the pre-LoC virus contained all described optimal epitopes for his HLA class I alleles in wild-type immunogenic sequence, yet the subjects only mounted these Gag responses upon uncontrolled superinfection (17).

### Reduced antiviral capacity of CD8^+^ T cells after loss of virological control.

**FIG 5:**
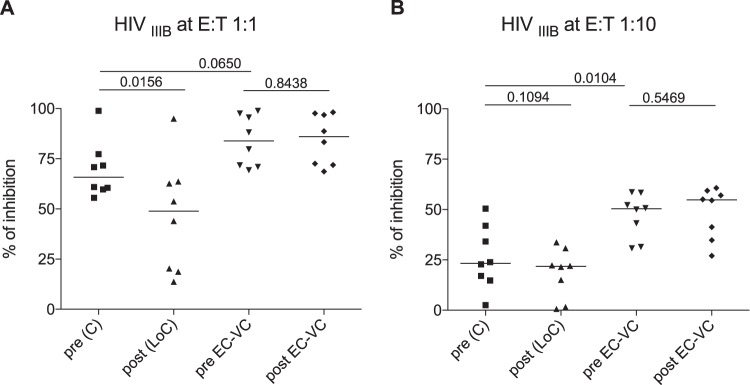
Viral inhibition capacity. Levels of CD8^+^ viral inhibitory capacity are shown for IIIB E/T 1:1 (A) and E/T 1:10 (B) in individuals who presented a loss of control (*n* = 8) and long-term controllers (*n* = 8). Comparison of antiviral capacity is expressed as % inhibition = [(fraction of p24^+^cells in CD4^+^ T cells cultured alone) − (fraction of p24^+^cells in CD4^+^ T cells cultured with CD8^+^ T cells)]/(fraction of p24^+^ in CD4^+^ T cells cultured alone) × 100.

*In vitro* inhibition of viral replication by autologous CD8^+^ T cells has become a standard measure to determine the *ex vivo* antiviral activity of cytotoxic T lymphocyte responses. We asked whether the viral inhibition assay (VIA) activity in individuals suffering an abrupt loss of control would be reduced at pre-LoC in comparison to persistent elite and viremic controllers and how VIA activity would change after LoC. In the absence of autologous virus isolates, the HIV IIIB laboratory strain was used to test *in vitro* inhibitory capacity of PBMC-derived CD8^+^ T cells. As a comparison, isolated CD8^+^ T cells from longitudinal PBMC samples of eight long-term HIV controllers with persistent *in vivo* HIV control (*n* = 6 EC and *n* = 2 VC, >15 years of diagnosed HIV-2 infection; longitudinal samples were 102 [range, 45 to 156] months apart between both follow-up time points) were tested (Table S1). Viral replication was measured by flow cytometry as a percentage of HIV Gag p24-positive cells in culture. The results showed a strong capacity of CD8^+^ T cells from pre-LoC samples to inhibit HIV replication at a 1:1 E/T (effector-to-target cells) ratio. However, this activity at the 1:1 ratio among the LoC patients studied here was somewhat lower than that of HIV controllers (median, 65% of LoC individuals compared to 84% of controllers, *P* = 0.0650; Fig. 5A) and significantly weaker at the 1:10 E/T ratio (median, 23% of LoC compared to 50% of controllers, *P* = 0.0104; Fig. 5B). While virus-suppressive activity in long-term controllers was maintained over time (*P* = 0.8438; median, 102 months between both time points tested), the suppressive capacity in LoC further decreased, especially detectable at the 1:1 ratio (*P* = 0.0156, Fig. 5A) upon loss of control. Thus, the data suggest that declining VIA activity over pre-LoC time points may be a prognostic marker for an increased risk of LoC.

### Elevated frequencies of CD38 and PD-1 expression in CD8^+^ T cells after LoC.

**FIG 6:**
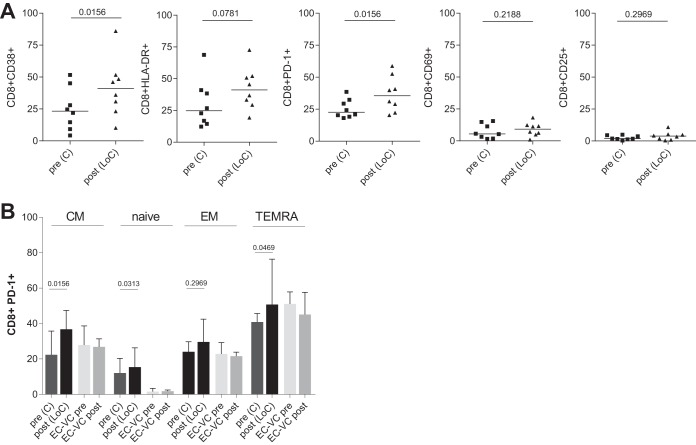
Levels of activation and exhaustion markers. (A) Percentage of expression of CD38, HLA-DR, PD-1, CD25, and CD69 on total CD8^+^ T cells in individuals that experience a loss of control (*n* = 8) tested before [pre (C)] or after [post (LoC)] loss of viral control. Values are compared to staining in samples from persistent HIV controllers taken at a median of 102 months apart. Markers were assessed following the gating strategy shown in Fig. S1. *P* values are shown for Wilcoxon signed rank test. (B) Levels of expression of PD-1 in subsets of CD8^+^ T cells populations based on CCR7 and CD45RA expression. Median and interquartile range values are shown for a group of 8 individuals that loss of control compared to 8 individuals maintained control over time. The gating strategy used is shown in Fig. S1. *P* values are shown for Wilcoxon signed rank test.

The expression of CD38 and HLA-DR on T cells has been shown to be lower in treated HIV-infected individuals under cART and in HIV controllers than in individuals with progressive HIV disease. We therefore asked whether individuals experiencing LoC had elevated levels of activated cells right before the loss of control and how the increased virus replication post-LoC may impact CD38 and HLA-DR expression. The expression of surface activation markers on CD4^+^ and CD8^+^ T cells was assessed by flow cytometry before and after a loss of HIV control, along with additional activation markers (HLA-DR, CD38, CD25, and CD69) and an exhaustion marker (PD-1). Longitudinal samples from persistent HIV controllers were tested for comparison (102 months apart; range, 45 to 156 months). The expression of activation markers pre-LoC was similar to that in long-term controllers (data not shown); therefore, there was no evidence for elevated residual viral replication in LoC that could drive higher levels of activated T cells. In contrast, an almost 2-fold increase in the percentage of CD38^+^ and a trend toward higher levels of HLA-DR^+^ CD8 cells were observed after a loss of control (*P* = 0.0156 for CD38^+^, *P* = 0.0781 for HLA-DR, and *P* = 0.1563 for HLA-DR^+^ CD38^+^). No alterations in the expression of CD25 or CD69 were observed over time in either CD8^+^ (Fig. 6A) or CD4^+^ T-cell populations among LoC individuals and did not differ in expression levels observed in long-term controllers (data not shown). In line with previous reports (26), CD8^+^ T cells also expressed higher levels of PD-1 post-LoC (*P* = 0.0156, Fig. 6A), especially in central memory cells (CD8^+^ CCR7-CD45RA^+^), while those levels were maintained in persistent controllers (Fig. 6B). Of note, there was a significantly higher level of PD-1^+^ CD8^+^ T cells with a naive phenotype (CCR7^+^ CD45RA^+^) in the individuals with LoC than in persistent controllers. Importantly, this difference was already manifest before LoC, potentially identifying an additional risk marker for subsequent LoC.

## DISCUSSION

The search for reliable predictors of HIV control has been largely focused on cross-sectional comparisons between individuals with either controlled or uncontrolled HIV infection in the absence of antiretroviral treatment. These studies have yielded a number of correlates of control, many pointing toward an important role of the virus-specific cellular immunity in the natural control of HIV (27–30). Interpretation of such studies, however, can be limited by its cross-sectional nature and, consequently, by significant potential differences in host genetics between comparison groups, in particular, HLA class I allele frequencies (8, 31). In the present study, we have attempted to overcome some of these limitations by analyzing longitudinal samples from subjects who spontaneously lose their previously excellent and longstanding control of viral replication. The identification of markers of control, or loss thereof, could be a helpful tool for the clinical management of individuals off cART with controlled HIV infection and could have important implications for future prevention and treatment strategies in general.

One possible explanation for the abrupt HIV disease progression observed in the present cohort could be HIV superinfection. Superinfection with HIV has been associated with disease progression (17, 32) and has been previously documented, even in the setting of HIV controller cohorts (33–35). Although superinfection can be facilitated by exposure to a virus that can escape preexisting virus-specific T-cell immunity (33, 36), the first well-documented case of an uncontrolled superinfection in an HIV controller occurred in the presence of a majority of T-cell epitopes being conserved between the first and second infecting virus (17). Interestingly, in the one case of suspected superinfection in the present cohort, we observed a complete change in the pattern of CD8^+^ T-cell responses, where only 2 responses were maintained (in Pol and Nef), while 15 new responses toward the whole proteome of HIV were mounted; however, they were evidently not able to control the new virus. Although limited by the cohort size, the present data are in line with earlier reports of highly immunogenic but uncontrolled superinfection and suggest that superinfection may not be a major factor in the sudden loss of virus control in former HIV controllers.

An alternative explanation for LoC could be a switch in the viral tropism from CCR5 to CXCR4, which has been described to precede a decrease in CD4 T-cell counts and progression to AIDS (37–40). We found that 30% of the individuals (4 out of 13 tested) presented X4-tropic virus after progression, while none was detected during the controlled stages of infection. Interestingly, one of the individuals that showed an X4-tropic virus was the subject that acquired superinfection, suggesting that the second viral infection may have been with an X4-tropic virus. For the remaining 3 subjects who harbored X4 virus post-LoC, it remains unclear whether the change of tropism was either the cause or the consequence of the loss of control. Actually, despite significant advances in the study of relevant parameters involved in HIV control, it is still difficult to define the causality dilemma of certain factors, such as the HIV tropism evolution (28). Although a switch in coreceptor usage has been attributed to an increase in viral fitness (41, 42), we were unable to obtain any autologous virus from samples drawn at time points when the individuals were controlling the infection and thus could not compare viral replicative fitness pre- and post-LoC. Cell tropism of HIV has also been associated with cell type-specific differences in antigen processing and, hence, differences in epitope presentation on infected T cells versus infected macrophages or dendritic cells (43). An R5-to-X4 tropism change could therefore result in an effective escape from T-cell immunity that is mainly focused on epitopes presented by infected T cells but not by macrophages. In addition, it could also explain the marked increase in responses post-LoC in the subject with superinfection and that *env* responses were only seen after superinfection, although the epitopes were present in wild-type sequence in the controlled virus pre-LoC. As only three individuals showed evidence of a dominant X4-tropic virus post-LoC, we were not able to identify any specific response pattern that could correlate with tropism change during HIV infection progression.

As referred to above, HIV controller cohorts are oftentimes enriched in particular “beneficial” HLA class I alleles, including HLA-B*27, HLA-B*57, and others (6). Our cohort of LoC is no exception to this, as the frequency of those protective HLA class I alleles (B57/58 and B27) was the same as observed in a locally recruited elite and viremic controller cohort (44). Furthermore, we also detected broad and strong CTL responses, especially toward Gag, consistent with previous reports in comparable cohorts (2, 45–48). However, it has also been suggested that HIV controllers with beneficial or nonbeneficial HLA class I alleles may control HIV through different mechanisms, with subjects expressing nonbeneficial alleles, such as HLA-B*35, depending critically on their T-cell responses to durable control (44). As our cohort only included one subject with a strong nonbeneficial HLA class I alleles, the study of larger cohorts of LoC with unfavorable HLA class I genetics will offer a unique opportunity to determine whether the failures of these responses are indeed critical determinants in LoC. In addition, more in-depth characterization of these responses, including avidity testing and effector function profiling (49, 50), rather than its mere presence/absence pre- and post-LoC, may reveal factors that define the “failure” of these responses post-LoC. We addressed the measurement of functional activity here by conducting *in vitro* inhibition assays (VIA) on samples pre- and post-LoC. Our results indeed showed decreased antiviral *in vitro* capacity of CD8^+^ T cells before LoC compared to persistently controlling EC. Still, the data suggest that declined VIA activity may precede a loss of control and could serve as a predictor of failing immune control. This is in line with cross-sectional analyses that have associated antiviral capacity of CD8 T-cell in VIA with the rate of HIV disease progression and CD4^+^ T-cell decline (13, 24). Thus, monitoring VIA activity in untreated individuals may be a useful tool to initiate cART in a timely manner and may be especially useful in treatment interruption studies that assess outcomes of therapeutic vaccination and other HIV cure approaches.

Another well-accepted explanation for loss of effective immune control of viral replication *in vivo* is the occurrence of CTL escape mutations in the targeted epitope. Although most tested individuals presented an increased number of HLA-associated escape mutation in their Gag sequence between pre- and post-LoC samples, the differences in HLA footprints over time were not significant. As a single escaped CTL epitope may allow for a loss of control, it would be important to test epitope variants for their true ability to escape CTL recognition. However, we did not have sufficient samples at hand to map all responses and assess the effects of epitope mutations on CTL recognition. In addition, extended deep sequencing covering the entire viral genome and additional sampling time points close to LoC would provide more insights into the role that HLA-associated mutations outside Gag may play in the abrupt the loss of control. Deep sequencing would also reveal low-frequency mutations present that were missed by our analyses and, with additional sampling points, allow the evolution and frequencies of these mutations to be closely followed. As the frequency of individuals experiencing a marked rapid LoC is already low, having closer sampling intervals in larger cohorts will be truly challenging. Due to technical hurdles and sample availability needed to reliably amplify *gag* sequences in individuals with low or undetectable viremia, we cannot compare the occurrence and frequency of Gag HLA-associated escape mutations seen in our subjects with those in individuals with persistent control. However, existing data show that even in individuals with low levels of viral replication, Gag viral evolution can be detected (45, 51), suggesting that their presence does not necessarily need to lead to LoC.

In line with the loss of antiviral capacity and an increased viral replication *in vivo*, we observed a significant increase in the expression of markers of T-cell activation and exhaustion (in particular, CD38^+^ and PD-1), both of which have been linked to HIV disease progression (9, 46–48, 52–54). Of note, LoC individuals showed a significantly higher level of PD-1-expressing CD8^+^ T cells even before loss of control compared to individuals that maintained their viral control over time. This was especially marked for CD8^+^ T cells with a naive phenotype and may represent an early sign of LoC during which terminally differentiated T cells may reacquire a naive phenotype (55). These cells may not be able to cope with ongoing viral replication and immune activation pre-LoC, ultimately leading to full-blown loss of control and elevated viral loads. At the same time, the increased levels of exhaustion markers could also explain the reduced VIA activity seen pre-LoC. Moreover, the enhanced activation and effector phenotype of CD8^+^ T cells could lead to less proliferative capacity of cells *in vitro* and result in reduced VIA activity seen post-LoC. If validated in further cohorts, our analyses may thus have identified reduced VIA activity and elevated levels of PD-1 expression as predictive markers of loss of control in individuals showing natural control of HIV and may help improve their clinical management. The data may also help guide immune-based therapeutic intervention to achieve a functional HIV cure by defining minimal VIA activities and preferred T-cell phenotypes that may warrant treatment interruptions.

In light of reports showing a predictive value of reservoir size and the rebound kinetics of virus after stopping treatment (56–58) or, in on our case, after LoC, it would have been interesting to determine the size of the viral reservoir in the LoC individuals tested here. However, cell availability did not allow for such analyses. Despite some of these limitations, the present report suggests that individuals who presented an abrupt loss of HIV control had an impaired capacity to inhibit *in vitro* viral replication, maybe due to the increased exhaustion of their CD8^+^ T cells before LoC. Measuring these parameters may help identify untreated HIV controllers that are at risk of losing control and may offer a useful tool for monitoring individuals during treatment interruption phases included in therapeutic vaccine trials or other cure strategies.

Earlier studies addressing loss of control in previous HIV controllers have also been hampered by the small size of the cohorts of such particular individuals or have attempted to address this question in simian immunodeficiency virus (SIV)-infected macaques (59, 60) while assessing only one or additional aspects of host immunity and their role in persistent virus control (28, 52, 61, 62). Among these, NK function (54), polyfunctionality (18), and the occurrence of new mutations in relatively conserved viral genes have been proposed to precede the abrupt loss of control. As in our study, these analyses are limited by the difficulty of discerning cause from effect of these events or mechanisms, and further work, possibly in SIV models, may be required to validate the importance of the individual factors identified in predicting LoC.

## MATERIALS AND METHODS

### Study participants and samples.

Among two existing cohorts of HIV-1-infected elite and viremic controllers (defined by sustained undetectable or low-level viremia, respectively [plasma viral load {pVL}, <50 copies/ml or <2,000 copies/ml] for more than 1 year in the absence of ART), we identified 14 subjects who experienced an abrupt transition from a nonprogressive to a progressive state of HIV infection (Table 1). All individuals had stable viremia at <3 log copies/ml before LoC for a median time of 14 years since HIV diagnosis (interquartile range [IQR], 4 to 19 years). The transition to LoC was defined as (i) >1-log pVL increase and/or (ii) loss of >30% CD4^+^ T-cell counts or drop below 350 CD4^+^ T cells/ml within 1 year. Samples were obtained from the Spanish HIVHGM BioBank supported by the Spanish Instituto de Salud Carlos III and from the existing long-term sample repository at IrsiCaixa, in Badalona, Spain. All subjects were HLA-typed for HLA-A, HLA-B, and HLA-C loci at high resolution, as described previously (31). Data from a full-protein ELISPOT CTL screen of a group of 65 individuals from the IrsiCaixa Controllers Cohort with persistent HIV control for a median time of 14 years since HIV diagnosis (IQR, 5 to 21 years) were included for comparisons and included 38 EC (median viral load, 50 copies/ml; median CD4 count, 815 cells/mm^3^) and 27 viremic controllers (median VL, 545 copies/ml; median CD4 count, 616 cells/mm^3^). A subset of eight HIV-1 controllers with longer clinical follow-up and with prospective biological samples available at separated time points was selected for the viral inhibition assay comparison as long-term controllers (Table S1). All patients provided informed consent before providing samples. The study was approved by the institutional ethical review board of the Hospital Universitari Germans Trias i Pujol (reference no. EO-09-041).

### Gag sequence evolution.

HIV-1 RNA was extracted from plasma using the QIAamp viral RNA kit (Qiagen) and was reverse transcribed using reverse transcription-PCR (RT-PCR) SuperScript III enzyme mix (Invitrogen). The *gag* gene region was then amplified by nested PCR. Sequencing was performed by the Genomics Core Facility at Germans Trias i Pujol Research Institute. Gag nucleotide alignment was performed with MUSCLE (http://www.ebi.ac.uk/Tools/msa/muscle/) to generate the corresponding multiple-sequence alignments (MSA). Nucleotide evolution models that best explained sequence evolution within each MSA were identified using the MEGA5 Find Best DNA/Protein Models (ML) function. The model with the lower score according to Bayesian information criteria implemented in MEGA5 was selected to construct phylogenetic trees. Gag sequences obtained were further analyzed to determine the number of HLA class I-associated immune escape mutations and were identified based on the individual’s HLA class I genotype and the HLA-associated escape mutations described by Brumme et al. (23).

### Determination of viral tropism.

HIV tropism was predicted by Gp160 V3-loop region Sanger sequencing, as previously described (63). Briefly, HIV-1 RNA was extracted from plasma using the QIAamp viral RNA kit (Qiagen), and cDNA was synthetized using RT-PCR SuperScript III enzyme mix (Invitrogen). The *env* V3-loop-encoding region was amplified by nested PCR. Sequences were analyzed by using the Geno2Pheno [coreceptor] using a false-positive rate (FPR) cutoff of 10% to define the presence of an X4 HIV-1.

### IFN-γ ELISPOT.

All individuals were screened longitudinally for IFN-γ-secreting T-cell responses to the entire HIV proteome. Briefly, cryopreserved PBMC were thawed and rested for 5 h at 37°C before plating 100,000 live cells per well in IFN-γ ELISPOT 96-well polyvinylidene plates (Millipore). PBMC were stimulated with a clade B consensus sequence set of 410 peptides (18mers overlapping by 11 amino acids [aa]) at a final concentration of 14 μg/ml. The IFN-γ Mabtech kit was used, according to the manufacturer’s instructions. Spots were counted using an automated ELISPOT reader system (ImmunoSpot S6 Versa; CTL, Germany), and the magnitude of responses was expressed as spot-forming cells (SFC) per million input cells. The threshold for positive responses was defined as at least 5 spots per well, responses exceeding the “mean number of spots in negative-control wells plus 3 standard deviations of the negative-control wells” and “three times the mean of negative-control wells,” whichever was higher.

### Viral inhibition assay.

The capacity of CD8^+^ T cells to suppress HIV-1 replication in autologous CD4^+^ T cells *ex vivo* was assessed as described in detail elsewhere (69, 70). Briefly, CD8-depleted cells were isolated from cryopreserved PBMC by magnetic bead separation (MACS Milteny Biotec) and stimulated with phytohemagglutinin (PHA; 5 μg/ml) for 3 days in R10-RPMI medium supplemented with 10% fetal calf serum (FCS), l-glutamine (2 mM), penicillin (100 U/ml), and streptomycin (100 μg/ml). Cells were then washed and infected by spinoculation with an HIV-1 IIIB strain at a multiplicity of infection (MOI) of 0.01. To assess the antiviral capacity of CD8^+^ T cells, autologous infected CD4^+^ T cells were cultured alone or with unstimulated CD8^+^ T cells in R10 supplemented with interleukin 2 (20 IU/ml). The experiments were carried out in triplicate at different effector-to-target ratios (1:1, 1:10, and 1:2, depending on cell availability). After 6 days of coculture, intracellular p24 production was measured by flow cytometry. Antiviral capacity was expressed as percentage of inhibition, determined as [(fraction of p24^+^ cells in CD4^+^ T cells cultured alone) − (fraction of p24^+^ in CD4^+^ T cells cultured with CD8^+^ cells)]/(fraction of p24^+^ cells in CD4^+^ T cells cultured alone) × 100.

### Flow cytometry.

CD8^+^-depleted cells (CD4^+^ T-cell enriched fraction) and CD8^+^-isolated T cells were stained for activation and exhaustion markers. Cells were first stained with a viability staining (Aqua LIVE/DEAD fixable dead cell stain kit; Invitrogen), followed by exclusion staining of B lymphocytes and myeloid cells using combined markers in a dump channel (using the cells/antibodies CD19/BV510 and CD14/BV510; BioLegend). For T-cell lineage and activation markers, the following cells/antibodies were used: CD3/antigen-presenting cells (APC) Cy7, CD4/peridinin chlorophyll protein (PerCP) (BD Biosciences); and CD8/PerCP, CCR7/BV421, CD45RAPECF594/HLA-DR fluorescein isothiocyanate (FITC), PD-1/phycoerythyrin (PE,) CD69/APC, CD38/BV785, and CD25/BV605 (BioLegend). After staining, cells were collected on an LSRFortessa instrument (BD), and analysis was performed using the FlowJo 10 software.

To determine the number of p24^+^ cells in the viral inhibition assay, cells were stained with Aqua LIVE/DEAD (Invitrogen), followed by extracellular staining with cells/antibodies CD3/APC-H7, CD4/PerCP, and CD8/APC (BD Biosciences). Cells were fixed and permeabilized (FIX & PERM cell fixation & cell permeabilization kit; Thermo Fisher Scientific) and finally stained with p24 antibody KC.57 fluorescein isothiocyanate (FITC; Beckman Coulter). Samples were acquired on an LSR-II cytometer, and data analysis was done using the FlowJo 10 software.

### Statistical analysis.

GraphPad Prism version 7.0 for Windows (San Diego, CA) was used to compare response rates in both groups and subgroup analyses. A Mann-Whitney test and Wilcoxon matched paired test were used for unpaired and paired comparisons, respectively.

### Data availability.

Gag sequences are available at GenBank with the accession numbers MK086511 (LP1), MK086512 (LP2 pre), MK086513 (LP2 post), MK086514 (LP3 pre), MK086515 (LP3 post), MK086516 (LP4 pre), MK086517 (LP4 post), MK086518 (LP5 pre), MK086519 (LP5 post), MK086520 (LP6 post), MK086521 (LP7 post), MK086522 (LP8 pre), MK086523 (LP8 post), MK086524 (LP9 pre), MK086525 (LP9 post), MK086526 (LP10 post), MK086527 (LP11 pre), MK086528 (LP11 post), MK086529 (LP12 pre), MK086530 (LP12 post), MK086531 (LP13 post), MK086532 (LP14 pre), and MK086533 (LP14 post).

## Supplementary Material

Supplemental file 1
