# Supplementary material for: Mechanisms of Abrupt Loss of Virus Control in a Cohort of Previous HIV Controllers
Source: J Virol. 2019 Feb 5;93(4):e01436-18. doi: 10.1128/JVI.01436-18 (PMC6363998; doi:10.1128/JVI.01436-18)
Supplement: Supplemental file 1 [file a43aa757b3205769f9a5736545cfa275_JVI.01436-18-s0001.pdf]

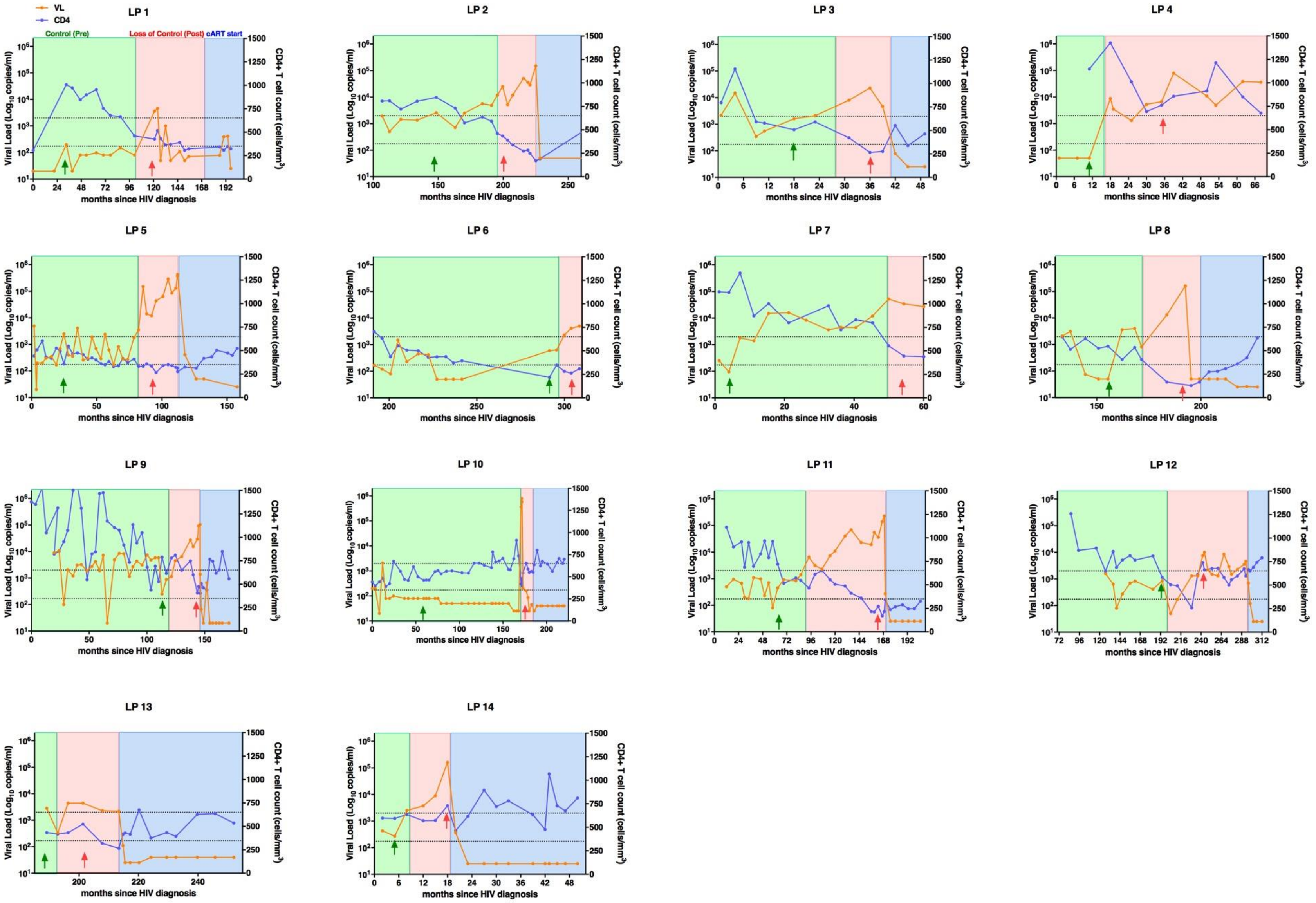


**Supplementary figure 1:** Evolution of viral load (orange lines) and CD4 counts (blue lines) of all individuals included in the study. Green area represents time with viral control, red area the loss of control and blue area the time during cART. Time points analyzed are marked with a green arrow for the time point with controlled infection and red for uncontrolled time point.

| | | | | | | | | | |
|---|---|---|---|---|---|---|---|---|---|
| HXB2 | MGARASVLSG | GELDRWEKIR | LRPGGKKKYK | LKHIVWASRE | LERFAVNPGL | LETSEGCRQI | LGQLQPSLQT | GSEELRSLYN | TVATLYCVHQ |
| LP1 post | ---------- | ----K----- | ---------- | ---------- | ---------- | -------K-- | -E----A--- | -----K---- | ---------K |
| LP2 pre | ---------- | -K--Q----- | ---------- | ---------- | ---------- | ---------- | ---------- | -----K---- | ---------- |
| LP2 post | ---------- | -K--Q----- | ---------- | ---------- | ---------- | ---------- | ---------- | -----K---- | ---------- |
| LP3 pre | ---------- | -K--K--R-- | ---------R | ---------- | ---------- | ---------- | ---------- | --------**F**- | -I-------- |
| LP3 post | ---------- | -K--K--R-- | ---------R | ---------- | ---------- | ---------- | ---------- | --------**F**- | -I-------- |
| LP4 pre | ---------- | -Q--K----- | -------S-- | ---------- | ---------- | ---------- | ------A--- | ---------- | ---V------ |
| LP4 post | ---------- | -K-----R-- | -------Q-**R** | ---------- | ---------- | --------K- | IE-------- | --------F- | ---------E |
| LP5 pre | ------I--- | ---------- | ---------- | ---L------ | --------S- | ----D--K-- | ---------- | -----K-V-- | -I------L- |
| LP5 post | ------I--- | ---------Y | -------**R**-- | ---L------ | ---------- | ---AD----- | ---------- | -----K---- | ---------R |
| LP6 post | ---------- | ---------- | ---------- | ---------- | ---------- | --------K- | ---------- | -----K---- | ---V------ |
| LP7 post | ---------- | ---------- | -------Q-R | ---------- | -----L---- | -------K-- | ---------- | --------**F**- | ---V-----R |
| LP8 pre | ------I--- | ---------- | -------**Q**-R | ---L------ | ---------- | -------K-- | IR-------- | ---------- | ---------- |
| LP8 post | ------I--- | ---------- | -------**Q**-R | ---L------ | ---------- | -------K-- | IR-------- | ---------- | ---------- |
| LP9 pre | ---------- | ----K----- | -------**Q**-R | ---------- | ---------- | -------KK- | -K----A--- | ---------- | -I-------- |
| LP9 post | ---------- | ----K----- | -------**Q**-R | ---------- | ---------- | -------KK- | -E----A--- | ---------- | -I-------R |
| LP10 post | ---------- | EK--S----- | ---------**R** | ---L------ | -----L--**D**- | ---A---Q-- | M-----A--- | -T------F- | ---V-F---K |
| LP11 pre | ---------- | -K--Q----- | ---------R | ---------- | ---------- | ---AG--A-- | -T-------- | -----K---- | ---------- |
| LP11 post | ---------- | -K--Q----- | ---------R | ---------- | ---------- | ---AK--A-- | -T-------- | -----K---- | -I-V------ |
| LP12 pre | ---------- | -K--Q-GE-- | ---------- | ---------- | ---------- | I--------- | ---------- | -----K---- | ---------- |
| LP12 post | ---------- | -K--Q----- | -----**R**---- | ---------- | ---------- | I--------- | ---------- | -----K---- | ---------- |
| LP13 post | ---------- | ---------- | --Q------- | ---------- | ---------- | ---A------ | ------A--- | ---------- | ---------K |
| LP14 pre | ---------- | ---------- | ---------- | ---------- | ------D--- | ---------- | IR-IP----- | --------F- | ---V------ |
| LP14 post | ---------- | ---------- | ---------- | ---------- | ------D--- | ---------- | IR-IP----- | --------F- | ---V------ |

```
HXB2      RIEIKDTKEA LDKIEEEQNK SKKKAQQAAA DTGHSNQVSQ NYPIVQNIQG QMVHQAISPR TLNAWVKVVE EKAFSPEVIP MFSALSEGAT
LP1 post  ---------- -E-L------ -------TT- --RN-S---- ---------- ------L--- ---------- ---------- ----------
LP2 pre   ---V------ -E-------- ----------AG--NNS---- ---------- ---------- ---------- ---------- ----------
LP2 post  ---------- -E-------- ----------AG--DNS---- -------L-- ---------- --------I- ---------- ----------
LP3 pre   G--V------ ---V------ ---------- N----S---- -------L-- ---------- --------I- ---------- --T-------
LP3 post  G--V------ ---V------ ---------- N----S---- -------L-- ------L--- --------I- ---------- --T-------
LP4 pre   ---VK----- ---------- ---------- -----S---- -------L-- ---------- ---------- ---------- ----------
LP4 post  K---R---D- ---------- ---------- -A--R----- -------L-- -----P---- --------I- ---------- --T-------
LP5 pre   ---------- ---------- ---------- ---------- ---------- ---------- ---------- ---------- ----------
LP5 post  ---------- ---------- ---------- ---------- ---------- ---------- ---------- ---------- ----------
LP6 post  K--------- ---------- ---------- ---------- ---------- ------L--- --------I- ---------- ----------
LP7 post  --DI------ ---------- ---S-K---T ---NNS---- ---------- ------L--- --------I- ---------- ----------
LP8 pre   K---R----- ---------- ---------- ---------- ---------- -----PL--- ---------- ---------- ----------
LP8 post  K---R----- ---------- I--------- ---------- ---------- -----PL--- ---------- ---------- ----------
LP9 pre   --D------- -D------- ---G------ ----------- ---------- ---------- ---------- ---------- ----------
LP9 post  --D------- -D------- ---------- ----------- ---------- ---------- ---------- ---------- ----------
LP10 post -K-------- -E-V-KA-K- GQE-Q-A-MD EGS  --A-- -------A-- ---------- ---------- ---------- ----------
LP11 pre  --DV--Q--- -E-------- ---------- -----S-A-- -------M-- ------L--- --------I- ---------- ----------
LP11 post --DV--Q--- -A-------- ---------- ---D-S-A-- -------M-- ------L--- --------I- ---------- --T-------
LP12 pre  K--V------ -E-------- ----- ---- -A-NNS---- -------LH- ------L--- --------I- ---------- ---------A
LP12 post K--V------ -E-------- ----- ---- -T-NNS---- -------L-- -----PL--- --------I- ---------- ---------A
LP13 post --I------- -E-------- ---------- --R------- --------I- ---------- ---------- ---------- ----------
LP14 pre  KV-------- -E-L----K- -QQMT----- -K-    --- -----K---- ------L--- --------I- ---------- ------D---
LP14 post KV-------- -E-L----KE -QQTT----- -K-    --- ---------- ------L--- --------I- ---------- ------D---
```

```
HXB2       PQDLNTMLNT VGGHQAAMQM LKETINEEAA EWDRVHPVHA GPIAPGQMRE PRGSDIAGTT STLQEQIGWM TNNPPIPVGE IYKRWIILGL
LP1 post   ---------- ---------- ---------- ---------- ---------- ---------- -N-----A-- ---------- ----------
LP2 pre    ---------- ---------- ---------- ---------- --V------- ---------- ---------- ---------- ----------
LP2 post   ---------- ---------- ---------- ---------- --V------- ---------- ---------- ---------- ----------
LP3 pre    ---------- ---------- --D------- ---------- --V------- ---------- ---------- -S-------- ----------
LP3 post   ---------- ---------- --D------- ---------- --V------- ---------- ---------- ---------- ----------
LP4 pre    ---------- ---------- ---------- ---------- ---------- ---------- ---------- ---------- ----------
LP4 post   ---------- ---------- ---------- ---------- --V------- ---------- -------A-- -----V---- ----------
LP5 pre    ---------- ---------- ---------- ---------- --V------- ---------- ---------- ---------- ----------
LP5 post   ---------- ---------- ---------- ---------- --V------- ---------- ---------- ---------- ----------
LP6 post   ---------- ---------- ---------- --------Q- --L------- ---------- -N-A--VA-- -G-------- ----------
LP7 post   ---------- ---------- ---------- ---------- ---------- ---------- -N-----A-- ---------- ----------
LP8 pre    ---------- ---------- ---------- D--------- ---------- ---------- ---------- ---------- ----------
LP8 post   ---------- ---------- ---------- D--------- ---------- ---------- ---------- -H-------- ----------
LP9 pre    ---------- ---------- ---------- ---------- --V------- ---------- ---------- ---------- -------M--
LP9 post   ---------- ---------- ---------- ---------- --V------- ---------- ---------- ---------- -------M--
LP10 post  ---------- ---------- --D------- ----M--PQ- ---P---I-- ---------- -N-----T-- ---------- ----------
LP11 pre   ---------- ---------- ---------- ---------- --V------- ---------- -N-------- ---------- ----------
LP11 post  ---------- ---------- ---------- --------Q- --V------- ---------- -N-------- -H-------- ----------
LP12 pre   ---------- ---------- ---------- ---------- --V------- ---------- ---------- ---------- ----------
LP12 post  ---------- ---------- ---------- ---------- --A------- ---------- ---------- ---------- ----------
LP13 post  ---------- ---------- ---------- ---------- ---------- ---------- ---------- ---------- ----------
LP14 pre   ---------S ---------- --D---D--- D---T----- ---P------ ---------- ---N---Q-- -S---V---- ----------
LP14 post  ---------S ---------- --D------- ----T----- ---P------ ---------- -N-N---Q-- -S---V---- ----------
```

| HXB2 | NKIVRMYSPT | SILDIRQGPK | EPFRDYVDRF | YKTLRAEQAS | QEVKNWMTET | LLVQNANPDC | KTILKALGPA | ATLEEMMTAC | QGVGGPGHKA |
|---|---|---|---|---|---|---|---|---|---|
| LP1 post | ---------- | ---------- | -S-------- | ---------- | ---------- | ---------- | ---------- | ---------- | ---------- |
| LP2 pre | -------C-V | ---------- | ---------- | -**R**-------- | ---------- | ---------- | ---------- | ---------- | ---------- |
| LP2 post | -------S-V | -----**K**---- | ---------- | --**V**------- | ---------- | ---------- | ---------G | ---------- | ------S--- |
| LP3 pre | ---------- | ---------- | ---------- | ---------- | -D-------- | ---------- | ---------G | ---------- | ---------- |
| LP3 post | ---------- | -----**K**---- | ---------- | ---------- | -D-------- | ---------- | ---------G | ---------- | ------**S**--- |
| LP4 pre | ---------- | -----K---- | ---------- | -R-------- | ---------- | -----S---- | ---------- | ---------- | ---------- |
| LP4 post | ---------V | ---------- | ---------- | ---------- | -**D**--A----- | ---------- | --------S**G** | -------S-- | ---------- |
| LP5 pre | ---------V | ---------- | ---------- | ---------- | ---------- | ---------- | ---------- | ---------- | ------S--- |
| LP5 post | ---------V | ---------- | ---------- | ---------- | ---------- | ---------- | -N-------- | ---------- | ---------- |
| LP6 post | ---------- | -----K---- | ---------- | ---------**T** | ---------- | ---------- | ---------- | ---------- | ------S--- |
| LP7 post | ---------V | ---------- | ---------- | ---------- | ---------- | ---------- | ---------- | ---------- | ---------- |
| LP8 pre | ---------V | -----K---- | ---------- | ---------- | -D-------- | ---------- | ---------- | ---------- | ---------- |
| LP8 post | ---------- | -----K---- | ---------- | ---------- | ---------- | ---------- | ---------- | ---------- | ---------- |
| LP9 pre | ---------- | -----K---- | ---------- | ---------- | ---------- | ---------- | ---------- | ---------- | ---------- |
| LP9 post | ---------- | -----K---- | ---------- | ---------- | ---------- | ---------- | ---------- | ---------- | ---------- |
| LP10 post | ---------V | ---------- | ---------- | ---------T | ----G---D- | ---------- | ----R----G | -S-------- | ---------- |
| LP11 pre | ---------V | ----V----- | ---------- | --I------- | -**D**-------- | ---------- | ---------- | ---------- | ---------- |
| LP11 post | ---------V | ----VK---- | ---------- | --I------- | -**D**-------- | ---------- | ---------- | ---------- | ---------- |
| LP12 pre | ---------V | ---------- | ---------- | ---------- | ---------- | ---------- | ---------- | ---------- | ---------- |
| LP12 post | ---------V | ---------- | ---------- | ---------- | ---------- | ---------- | ---------- | ---------- | ---------- |
| LP13 post | ---------**S** | ---------- | ---------- | ---------- | ---------- | ---------- | ---------- | ---------- | ---------- |
| LP14 pre | ---------V | ---------- | ---------- | F--------T | ---------- | ---------- | ---------- | ---------- | ---------- |
| LP14 post | ---------V | ---------- | ---------- | F--------T | ---------- | ---------- | ---------- | ---------- | ---------- |

```
HXB2        RVLAEAMSQV TNSA·TIMMQR GNFRNQRKIV KCFNCGKEGH TARNCRAPRK KGCWKCGKEG HQMKDCTERQ ANFLGKIWPS YKGRPGNFLQ
LP1 post    ---------- -G--A------ ---------- ---------- --R------- ---------- ---------- ---------- ----------
LP2 pre     ---------- -S--·N----- ---------- ---------- I--------- ---------- ---------- ---------- H-------H-
LP2 post    ---------- -S--·NV---- ----G---S- ---------- I--------- ---------- ---------- ---------- H-------H-
LP3 pre     ---------- --TP·N----- --Y------  ---------- L-K------- ---------- ---------- ---------- NR--------
LP3 post    ---------- --TP·N----- --Y------  ---------- L-K------- ---------- ---------- ---------- HR--------
LP4 pre     ---------- ----·-V---- ---------- ---------- ---------- ---------- ---------- ---------- -R--------
LP4 post    ---------- ----·-----G ---K--G-T- -------V-- --K------- ---------- ------I--- ------V-S- -R--------
LP5 pre     ---------- ----·-V---- ---------- ---------- I-R--K---- R--------- ---------- ---------- ----------
LP5 post    ---------- ----·-V---- ---------- ---------- I-R--K---- R--------- ---------- ---------- ----------
LP6 post    ---------A ----·-V---- ----S----- ---------- ---------- R--------- ---------- ---------- ----------
LP7 post    ---------- ----·-----K --S-----SI ------R--- ---------- ---------- ---------- ---------- HR--------
LP8 pre     ---------- ----·------ ---R------ ---------- I-R------- ---------- ---------- ---------- ----------
LP8 post    ---------- ----·------ ---R------ ---------- I-R------- ---------- ---------- ---------- ----------
LP9 pre     ---------- -S--·------ --------M- ---------- I-R------- ---------- ---------- ---------- ----------
LP9 post    ---------- ----·------ --------M- ---------- I-R------- ---------- ---------- ---------- ----------
LP10 post   ---------A - K ·AT---K S--KGP-RQI ---------- L--------- ---------- ---------- ---------- H---------
LP11 pre    ---------- ---P·------ ---------- ---------- I--------- R--------- ---------- ---------- --------P-
LP11 post   ---------- ---S·A----K ---------- ---------- I--------- R--------- ---------- ---------- --------P-
LP12 pre    ---------- ---S·------ ---------- ---------- I-R------- ---------- ---------- ---------- --------P-
LP12 post   ---------- ---S·------ --------P- ---------- I-R------- ---------- ---------- ---------- --------P-
LP13 post   ---------- ----·------ ---------- ------R--- ---------- ---------- ---------- ---------- H---------
LP14 pre    ---------- -STN· ----- S--KS--RM- -------D-- ---------- ---------- ---------- ---------- N---------
LP14 post   ---------- -STN· ----- S--KS--RM- -------V-- ---------- ---------- ---------- ---------- N---------
```

| HXB2 | SRPEPTAPPE | ESFRSGVETT | TPPQKQEPID | KELYP·LTSLR | SLFGNDPSSQ |
|---|---|---|---|---|---|
| LP1 post | G--------- | ----F-E--- | --S---GQ-- | ---S-·-A--- | ---------- |
| LP2 pre | --L------- | ----F-E--- | --S------- | -----·----- | ---------- |
| LP2 post | --L------- | ----F-E--A | ---------- | --M--·-A--- | ---------- |
| LP3 pre | -----P---- | ----F-E--- | --S---GQ-- | --V--·----- | ---------- |
| LP3 post | -----P---- | ----F-E--- | --S---GQ-- | --V--·----- | ---------- |
| LP4 pre | ---------- | ---GF-G--- | --S------- | --N--·----- | ---------- |
| LP4 post | N--------- | ----F-E--- | --S----T-- | --K--·----- | ---------- |
| LP5 pre | ---------- | ---------P | --S------- | -----·----- | ---------- |
| LP5 post | ---------- | ---------P | --S------- | -----·----- | ---------- |
| LP6 post | --L------- | --------M- | --S------- | -----·----- | ---------- |
| LP7 post | ---------- | ----F-E--- | --S-----K- | Q-K--·-A--- | ---------- |
| LP8 pre | N--------- | ----F-E--- | --S------- | -----·----- | ---------- |
| LP8 post | N--------- | ----F-E--- | --S------- | -----·----- | ---------- |
| LP9 pre | ---------- | ----F-E--A | --S------- | -----·----- | ---------- |
| LP9 post | ---------- | ----F-E--A | --S------- | -----·----- | ---------- |
| LP10 post | ---------A | ---GF-E-IA | P- L---TKK | E----·-A--K | ----S----- |
| LP11 pre | --L------- | ----F-E--- | --SR-P-**L**-- | -D-S-·-A--- | ---------- |
| LP11 post | --L------- | ----F-EG-- | --SR-P-**L**-- | -D-S-·-A--- | ---------- |
| LP12 pre | --L------- | ----F-E--- | --S------- | --M--·----- | ---------- |
| LP12 post | --L------- | ----F-EG-- | --S------- | --M--·----- | ---------- |
| LP13 post | --L------- | ------E--- | --S------- | ---H-·----- | ---------- |
| LP14 pre | N--------A | ---GF-E- I | ---P---QKT | EG---P-A--- | R--------- |
| LP14 post | N--------A | ---GF-E- I | ---P---QKT | EG---P-A--- | R--------- |

**Supplementary figure 2:** Alignment of Gag sequences obtained for each individual (pre and post LoC) to HXB2 sequence. HLA-associated footprints are marked in read.

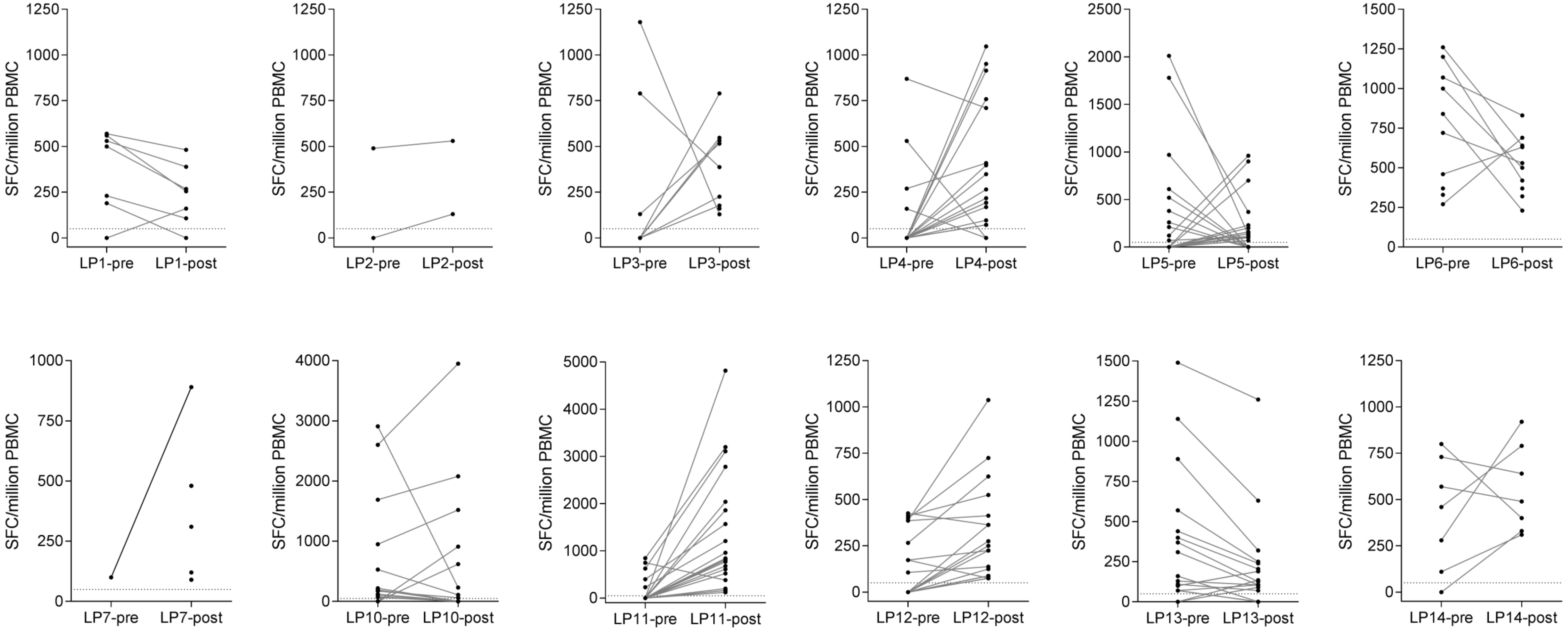


**Supplementary Figure 3:** Individual HIV-specific responses. Magnitude of CD8+ T cell HIV-specific responses expressed as SFC/ million PBMC is shown longitudinally before and after progression (n=12).

**Supplementary table 1**: Clinical characteristics of patients with persistent HIV control (long-term controllers).

| ID (EC/VC) | Age, years* | Gender | Time since known HIV-1 infection (years)* | Log10 of viral load at 1st Follow-up timepoint studied | $Log_{10}$ of viral load at 2nd Follow-up timepoint studied | CD4 cells/mm$^3$ at 1st Follow-up timepoint studied | CD4 cells/mm$^3$ at 2nd Follow-up timepoint studied | HLA A | HLA B | HLA C | HLA DRB | HLA DQB |
|---|---|---|---|---|---|---|---|---|---|---|---|---|
| VC-2515 | 49 | Male | 16 | UD | 2,95 | 777 (29%) | 979 (29%) | 0101/3201 | 4002/5701 | 0202/0602 | 0701/1301 | 0303/0603 |
| VC-9540 | 63 | Female | 27 | UD | 2,83 | 529 (25%) | 503 (29%) | 0101/2902 | 4403/5701 | 0401/0602 | 0701/1101 | 0202/0301 |
| EC-1668 | 42 | Female | 15 | UD | UD | 1201 (40%) | 785 (44%) | 0201/0205 | 3905/5701 | 0602/1203 | 1301/1601 | 0502/0609 |
| EC-2143 | 55 | Female | 20 | UD | UD | 717 (33%) | 454 (27%) | 1101/2402 | 3501/5201 | 0401/1202 | 1301/1502 | 0601/0603 |
| EC-3901 | 47 | Female | 17 | UD | UD | 1201 (44%) | 689 (37%) | 0101/0201 | 0801/5701 | 0602/0701 | 0301/0701 | 0201/0303 |
| EC-1530 | 50 | Male | 28 | UD | 1,68 | 564 (22%) | 706 (31%) | 0201/3303 | 1302/5801 | 0302/0602 | 0701/1302 | 0202/0609 |
| EC-9079 | 55 | Female | 24 | UD | 2,30 | 645 (43%) | 450 (36%) | 0301/1101 | 0702/2705 | 0102/0702 | 0301/1101 | 0201/0301 |
| EC-9080 | 53 | Male | 22 | UD | UD | 440 (25%) | 493 (29%) | 0205/3101 | 4901/5101 | 0701/1502 | 1201/1301 | 0301/0603 |

**Age and HIV duration is shown for the latest timepoint analyzed. EC: elite controller. VC: viremic controller*
